# Controlled Ovarian Stimulation Using Medroxyprogesterone Acetate and hMG in Patients With Polycystic Ovary Syndrome Treated for IVF: A Double-Blind Randomized Crossover Clinical Trial

**DOI:** 10.1097/MD.0000000000002939

**Published:** 2016-03-07

**Authors:** Yun Wang, Qiuju Chen, NingLing Wang, Hong Chen, Qifeng Lyu, Yanping Kuang

**Affiliations:** From the Department of Assisted Reproduction, Shanghai Ninth People's Hospital, JiaoTong University School of Medicine, Shanghai, China.

## Abstract

Ovarian hyperstimulation syndrome (OHSS) during ovarian stimulation is a current challenge for patients with polycystic ovarian syndrome (PCOS). Our previous studies indicated that progestin can prevent premature luteinizing hormone (LH) surge or moderate/severe OHSS in the general subfertile population, both in the follicular-phase and luteal-phase ovarian stimulation but it is unclear if this is true for patients with PCOS.

The aim of the article was to analyze cycle characteristics and endocrinological profiles using human menopausal gonadotropin (hMG) in combination with medroxyprogesterone acetate (MPA) for PCOS patients who are undergoing IVF/intracytoplasmic sperm injection (ICSI) treatments and investigate the subsequently pregnancy outcomes of frozen embryo transfer (FET).

In the randomized prospective controlled study, 120 PCOS patients undergoing IVF/ICSI were recruited and randomly classified into 2 groups according to the ovarian stimulation protocols: hMG and MPA (group A, n = 60) or short protocol (group B, n = 60).

In the study group, hMG (150–225IU) and MPA (10 mg/d) were administered simultaneously beginning on cycle day 3. Ovulation was cotriggered by a gonadotropinreleasing hormone (GnRH) agonist (0.1 mg) and hCG (1000IU) when dominant follicles matured. A short protocol was used as a control.

The primary end-point was the ongoing pregnancy rate per transfer and incidence of OHSS.

Doses of hMG administrated in group A are significantly higher than those in the controls. LH suppression persisted during ovarian stimulation and no incidence of premature LH surge was seen in both groups. The fertilization rate and the ongoing pregnant rate in the study group were higher than that in the control. The number of oocytes retrieved, mature oocytes, clinical pregnancy rates per transfer, implantation rates, and cumulative pregnancy rates per patient were comparable between the 2 groups. The incidence of OHSS was low between the 2 groups, with no significant difference.

The study showed that MPA has the advantages of an oral administration route, easy access, more control over LH levels. A possible reduction in the incidence of moderate or severe OHSS with the MPA protocol should be viewed with caution as the data is small. Large randomized trials with adequate sample size remain necessary.

## INTRODUCTION

Women with polycystic ovarian syndrome (PCOS) having assisted reproductive technology represent a therapeutic challenge. Tonic hypersecretion of LH during the follicular phase, hyperandrogenemia, and increased intrafollicular androgens are thought to predispose to poor oocyte quality, low fertilization rates, and high miscarriage rates in women with PCOS.^1^ Moreover, women with PCOS undergoing IVF are at high risk of developing ovarian hyperstimulation syndrome (OHSS).^2^ Although the use of the antagonist protocol may reduce the incidence of moderate and severe OHSS, the included studies have shown that the risk is still high (20% in the antagonist vs 32% in the long agonist group).^3^ And the risk of 20% for OHSS in the antagonist protocol is with the standard hCG-trigger. Using agonist-trigger may reduce the OHSS risk, but a recent study shows that there was no significant difference of moderate or severe OHSS with the antagonist OHSS as compared with the long agonist group.^3^ A meta-analysis demonstrated that IVF in women with PCOS results in an increased cycle cancellation rate as compared with women without PCOS.^4^ Therefore, there is an unmet demand for alternative ways of ovulation induction with improved efficacy and decreased OHSS incidence.

Our previous work has shown that progesterone can prevent moderate/severe ovarian hyperstimulation syndrome in controlled ovarian stimulation in normal ovulatory women, both in the follicular-phase ovarian stimulation and luteal-phase ovarian stimulation.^5,6^ Subsequently, we have further found that medroxyprogesterone acetate (MPA) is an effective oral alternative for the prevention of premature LH surge in woman undergoing controlled ovarian hyperstimulation (COH), and the pregnancy outcomes from FET indicated that the embryos originating from MPA co-treatment with hMG regimen had similar developmental potential as the short protocol.^6^

Reduced endometrial receptivity in controlled ovarian stimulation cycles and improvements in vitrification now make FET a viable alternative to fresh embryo transfer.^7^ In fact, the transfer of cryopreserved-thawed embryos in the embryo freeze-all protocol has been shown to result in improved pregnancy and delivery outcomes.^8–10^ One important advantage of new protocol HMG/MPA in our study is—GnRHa and low dose of hCG (1000IU) could be used to trigger ovulation and avoid early onset OHSS due to administration of lower dose of hCG triggering ovulation. And cryopreservation of all embryos with transfer in a subsequent cycle can largely decrease late onset OHSS. Besides this, the LH-suppression effects of progestin and efficacy of the freeze-all protocol suggest that progestin cotreatment may be used as an optimal regimen for PCOS patients who are undergoing In vitro fertilization/ Intra cytoplasmic sperm injection (IVF/ICSI) treatments.

We used MPA as an alternative to progestin for its unique advantages. MPA has moderate to strong progestin action, less androgenic properties, and does not interfere with measurement of endogenous progestin production.^11^ Therefore, the present study was undertaken to analyze cycle characteristics and endocrinological profiles using hMG in combination with MPA for PCOS patients who are undergoing IVF/ICSI treatments and investigate the subsequently pregnancy outcomes of FET.

## MATERIALS AND METHODS

### Study Setting and Patients

All cycles were performed at the Department of Assisted Reproduction of Shanghai Ninth People's Hospital, Shanghai Jiaotong University School of Medicine, from January 2014 to October 2014. The study protocol was approved by the Ethics Committee (Institutional Review Board) of Shanghai Ninth People's Hospital. The trial was registered with the Chinese Clinical Trial Registry (ChiCTR-OCH-14004424). It was conducted according to the Declaration of Helsinki for medical research. All participants provided informed consent after counseling for infertility treatments and routine IVF procedures.

### Inclusion Criteria

Patients with PCOS were included. The major criteria for diagnosis of PCOS were oligo- and/or anovulation, clinical or biochemical signs of hyperandrogenism and polycystic ovaries; these criteria are in accordance with the revised 2003 Rotterdam criteria for PCOS. Additional inclusion criteria were: age 18 to 39 years, no endometriotic cyst present, as assessed by transvaginal ultrasound examination. Patients with known previous poor ovarian response were excluded.^12^

### Exclusion Criteria

The exclusion criteria were: (1) clinically significant systemic disease such as renal failure; (2) endometriosis grade 3 or higher; (3) documented ovarian failure including basal FSH above 10 IU/L; (4) received hormonal treatments in the previous 3 months; (5) any contraindications to ovarian stimulation treatment.

### Allocation and Sample Size Estimate

This is a prospective noninferiority trial. No previous study had reported the efficacy of the combination of MPA and hMG in the PCOS patients. In our clinic, the mean number of oocytes of short protocol was about 15 and the standard deviation was 6 in the short protocol. A sample size of 58 in each group has a power of 0.85 at 5% significance to detect a difference of 2 in the number of oocyte retrieval. Given the possibility of dropouts, we designed the study to include a total of 60 women in each group.

### Randomization and Masking

Investigators randomly assigned the volunteers who accomplished inclusion criteria to 1 of the 2 groups in alternating fashion. Odd-numbered participants were allocated to the MPA group and even-numbered participants were assigned to the short protocol.

Study investigators, research coordinators, and participants were blind to intervention group assignments.

### Ovarian Stimulation and Embryo Culture

Patients in the study group were given hMG (Anhui Fengyuan Pharma ceutical Co, China) at a dose of 150 to 225 IU/day and MPA (Beijing Zhong Xin Pharmaceutical, China) 10 mg/day from day 3 of menstruation, after ultrasound screen and blood test confirmed the presence of a baseline hormone profile. The 150 IU daily hMG dose was used for patients with high antral follicle count (AFC) > 20 or slightly elevated basal follicle stimulating hormone (FSH) (7–10 IU/L), whereas a daily dose of 225 IU hMG was used for all other patients. Follicular monitoring started on menstruaion cycle day (MC) 7 to 8 and was performed every 2 to 3 days using a transvaginal ultrasound examination to record the number of developing follicles. Serum FSH, LH, E_2_, Progesterone, and Testerone concentrations were measured using patient blood tests on the same days as the ultrasound exams. The dose of hMG was adjusted according to oestradiol concentrations and ovarian response. Our previous work showed that cotriggering by GnRHa 0.1 mg and a low dose of hCG (1000 IU) had a more beneficial effect on oocyte maturation than triggering by GnRHa alone in the MPA cotreatment with gonadotropin in the general infertile women.^6^ So when 3 dominant follicles reached 18 mm in diameter, the final stage of oocyte maturation was cotriggered by Decapeptyl (0.1 mg) (Ferring International Center SA, Germany) and hCG 1000IU (Lizhu Pharmaceutical Trading Co, China).

A short protocol was used for the control group. Patients were administrated Decapeptyl 0.1 mg daily beginning on MC2. hMG were given at a dose of 150 to 225 IU/day from day 3 of menstruation and Decapeptyl continued until the day of hCG administration. A daily dose of HMG 150IU was administered to patients with high AFCs (>20) or slightly elevated basal FSH (7–10 IU/L), whereas the other patients were administered a daily dose of hMG 225IU. After 4 to 5 days, the ultrasound examination and serum hormone level tests were performed and the dose of hMG was adjusted according to oestradiol concentrations and follicle development. When dominant follicles reached 18 mm in diameter, 2000IU of hCG was administered for triggering.

Transvaginal ultrasound-guided oocyte retrieval was performed 36 to 37 hours after the triggering. Oocytes were fertilized using either conventional IVF or ICSI depending on the semen parameters. Examination of embryo quality included the number/uniformity of blastomeres and the degree of fragmentation. Embryo morphology was scored according to the criteria of Cummins.^13^ OHSS was defined according to previously published classification system.^14^

All the highest quality embryos (including at least 6 blastomeres and fragments < 50%) were frozen by vitrification on the third day after oocyte retrieval. The embryos that were not of top quality were placed for further extended culture until the blastocyst stage. During this stage, only good morphology blastocysts were cryopreserved. The procedure of freezing and thawing cleavage-stage embryos and blastocysts has been described elsewhere.^15^ Thawed embryos were considered to have survived if ≥50% of the blastomeres were intact.^16^ Only survived embryos would be transferred.

### Transfer of Cryopreserved-Thawed Embryos

We used letrozole and, if necessary, hMG to stimulate monofollicular growth. The common method used was as follows: letrozole 5 mg was administered from cycle day 3 to 7, and then follicle growth was monitored beginning on day 10. At times treatment included a low dose of hMG (75 IU/day) to stimulate follicular and endometrial lining growth. Administration of 5000 IU of hCG and the timing of FET were performed as described elsewhere.^5^ Natural cycle is few since most PCOS cases have irregular menstrual cycles or anovulation.

The hormone substituted cycle was performed for patients with thin endometria during stimulation cycles with daily oral administration of (EE; Shanghai Xinyi Pharmaceutical) 75 mg/day from day 3 to attain the criteria of endometrial thickness ≥8 mm. At that time, patients were given 0.4 g progestin (Laboratoires Besins-Iscovesco) intravaginal and estradiol valerate (Abbott Biological B.V, Netherland) 8 mg daily. The maximum number of transferred embryos was 2 per patient. The progesterone supplementation was continued until 10 weeks of gestation after pregnancy was achieved.

### Outcome Measures

The primary outcome measure was the ongoing pregnancy rate per transfer. Clinical pregnancy and ongoing pregnancy were considered as the presence of a gestational sac with fetal heart activity, as assessed by ultrasound at 7 weeks and 12 weeks of gestation, respectively.

Secondary outcome included the number of cumulus-oocyte complexes (COCs) retrieved, number of mature oocytes (MII oocyte) retrieved, fertilization rate, incidence of OHSS, duration of gonadotropin stimulation, total dose of hMG, cycle cancellation rate, and cumulative pregnancy. Cumulative pregnancy was defined as the total number of pregnancy using multiple frozen-thawed embryo transfers.

### Hormonal Measurement

Serum FSH, LH, E_2_, progesterone, and testerone were collected on day 3 of the stimulation cycle, cycle day 7 to 8 (after 4–5 days of stimulation), cycle day 9 to 11 (after 6–8 days of stimulation), the trigger day, and the day after trigger (∼12 h later after the injection of GnRHα and hCG). Hormonal levels were measured with chemiluminescence (Abbott Biologicals B.V, Netherlands). The upper limit of E_2_ measurement was 5000 pg/mL. The lower limits of sensitivity were as follows: FSH = 0.06 IU/L, LH = 0.09 IU/L, E_2_ = 10 pg/mL, P = 0.1 ng/mL, and T = 0.01 ng/mL.

### Statistical Analysis

Statistical analysis was performed by SPSS (version 16.0, SPSS Inc, Chicago, IL). The chi-squared test was used for categorical comparisons or Fisher's exact tests as appropriate. One-way ANOVA was used for continuous variables. The significant difference was considered at *P* value <0.05.

## RESULTS

### Patient Characteristics

**FIGURE 1:**
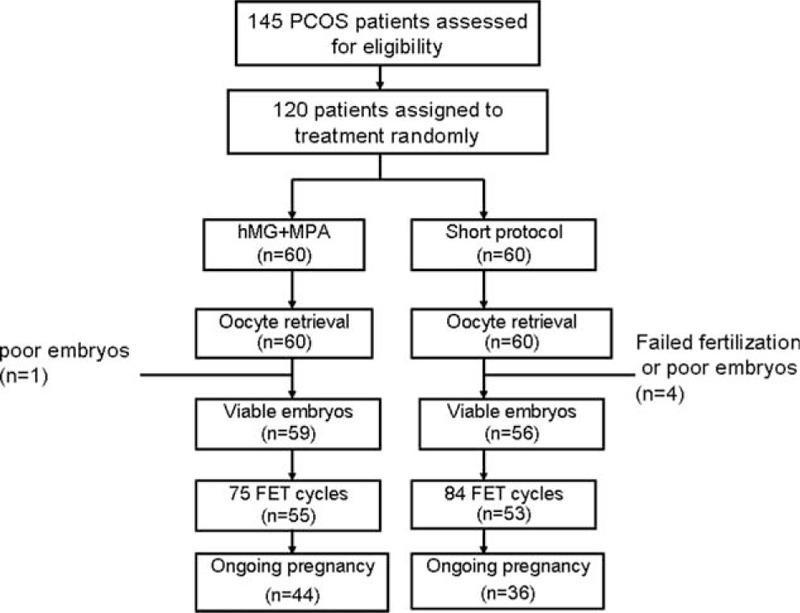
Study flow chart.

A total of 145 patients were assessed for eligibility; 120 patients were randomized into the 2 groups: MPA group (n = 60) and short group (n = 60). Figure 1 shows the flowchart of the study. A total of 120 women completed oocyte retrieval cycles and 159 completed FET cycles.

Baseline characteristics and hormonal profile of the patients analyzed are shown in Table 1. No significant differences were observed between the MPA and short group regarding the baseline characteristics, indication for IVF, previous IVF failures, and basal hormonal profile.

**TABLE 1:**
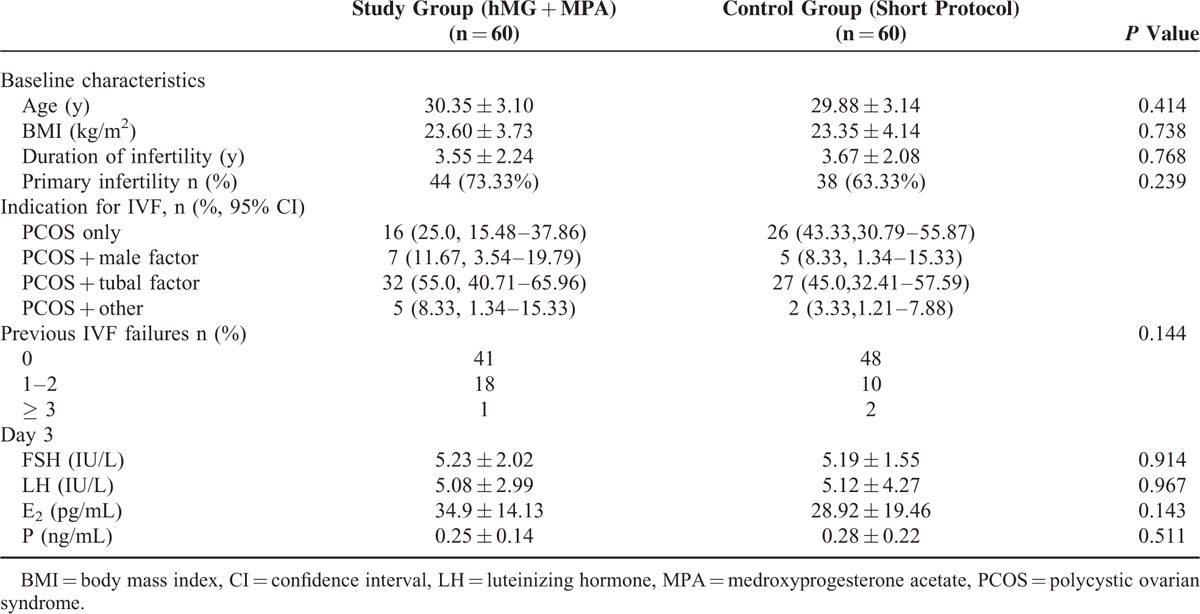
Baseline Characteristics and Hormonal Profile of Patients With PCOS in the Trial Undergoing IVF/ICSI Treatment

### Ovarian Stimulation, Follicle Development, and Oocyte Performance

The MPA group was characterized by a higher stimulation dose of hMG (2072.50 ± 467.86 vs 1501.25 ± 68.18, *P* < 0.05), but the stimulation duration showed no difference between the 2 groups (9.52 ± 2.01 vs 9.48 ± 3.33, *P* > 0.05). The number of follicles with diameters >10 mm (20.15 ± 8.09 vs 18.75 ± 9.61) or 14 mm (15.55 ± 8.83 vs 14.68 ± 9.58) were similar between the 2 groups. No significant differences were found in the oocyte retrieval rate, oocyte maturation rate, and cleavage rate between the 2 groups (*P* > 0.05). The fertilization rate was significantly higher in the study group (77.69 ± 16.59 vs 70.54 ± 19.23, *P* < 0.05). The number of top-quality embryos (4.98 ± 4.21 vs 5.00 ± 3.76) showed no significant difference between the 2 groups (*P* > 0.05). The cycle cancellation rate due to no viable embryos was not different between the 2 groups (1.67% vs 6.67%, *P* > 0.05). No patients experienced moderate or severe OHSS in the study group, but 2 patients in the control group developed moderate OHSS (Table 2).

**TABLE 2:**
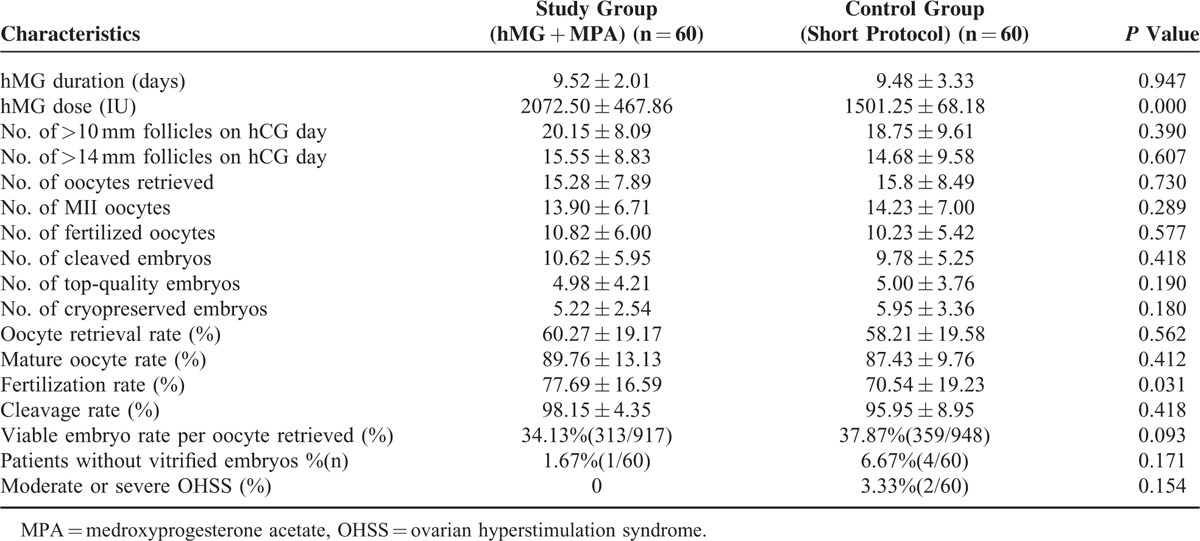
Ovarian Stimulation Characteristics and Hormonal Data on the Trigger Day in the 2 Regimens

### Hormonal Profile and Relevant Treatment

**FIGURE 2:**
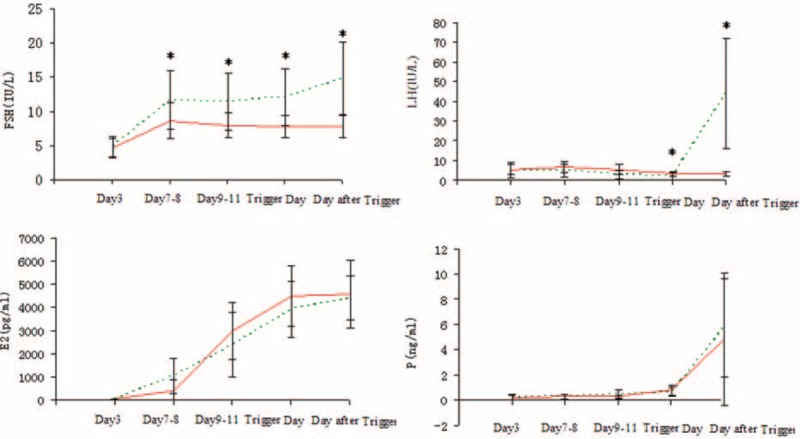
Serum hormone profiles present during ovarian stimulation in the 2 regimens. The green line refers to the MPA group and the red line stands for the control group. The asterisk (∗) represents *P* < 0.05 at the time point. MPA = medroxyprogesterone acetate.

Serum FSH, LH, E_2_, progesterone, and testerone in the 2 groups are shown in Figure 2. FSH levels increased after hMG administration in the study group (*P* < 0.05). After trigger, FSH increased to 15.09 ± 5.3 IU/L. There was a decreasing trend in serum LH values during ovarian stimulation and significant difference was found on basal levels compared with LH on trigger days (5.04 ± 4.13 vs 2.08 ± 1.82, *P* < 0.05). LH levels increased dramatically on the day after triggering (44.07 ± 27.84). E_2_ levels increased gradually accompanying follicles growth (*P* < 0.05). *P* showed a gradual increase during ovarian stimulation and increased again the day after ovulation triggering.

In the control group, FSH increased for about 3 to 4 days after hMG administration and then decreased slowly even after triggering. No incidence of LH surge or premature ovulation was found. E_2_ and P showed similar as the study group.

### Pregnancy Outcomes in FET Cycles

FET pregnancy outcomes originating from the 2 regimens are presented in Table 3. A total of 108 women across the 2 groups completed a total of 159 FET cycles, including 25 women who finished 2 FET cycles, 9 women who finished 3 FET cycles, and 3 women who finished 4 FET cycles. A total of 313 embryos were thawed and the rate of viable embryos after thawed was 99.68% (312/313). Both clinical pregnancy rate per transfer (65.33%) and implantation rate of embryos (48.65%) derived from the study group appeared higher than that from the control group (53.57%, 42.68%), but did not reach statistical significance (*P* > 0.05). Four women in the study group have miscarried in the first trimester (5.33%), whereas 8.33% (7/84) in the control group miscarried. The proportion of twin pregnancies (22.45% vs 17.78%) and ectopic pregnancy rate (2.04% vs. 2.22%) were similar between the 2 groups (*P* > 0.05). The ongoing pregnant rate was significantly higher in the study group than in the control group (58.67% vs 42.86%), which indicated embryos derived from the study group having better development potential. There was no significant difference of cumulative pregnancy rate between the 2 groups (80% vs 67.92%; *P* > 0.05).

**TABLE 3:**
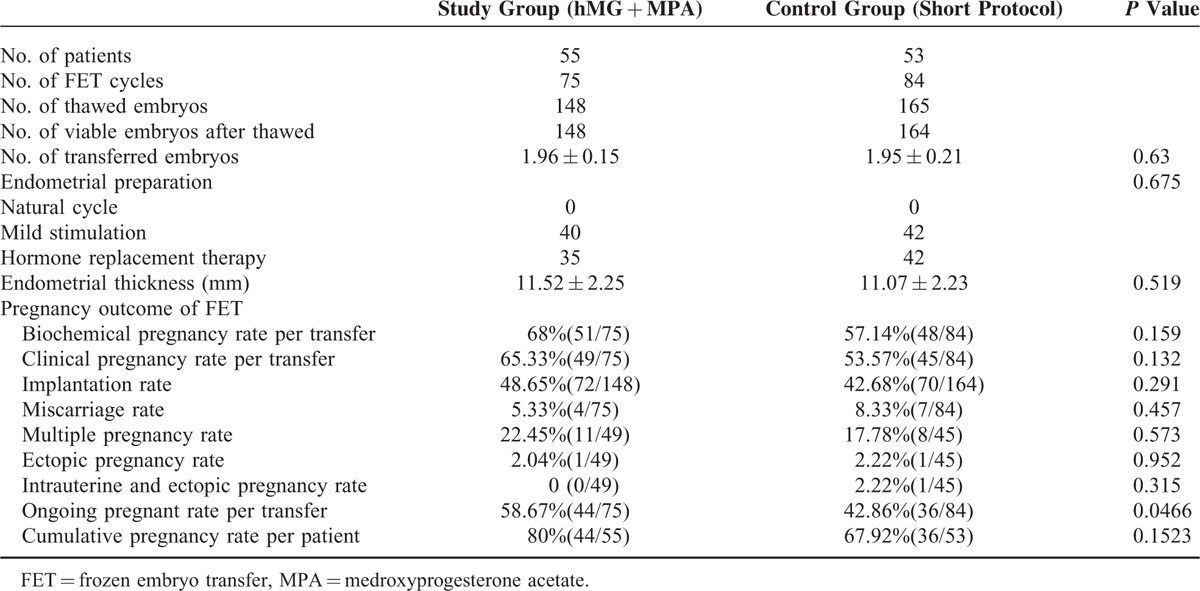
Pregnancy Outcomes of Frozen-Thawed Embryos Originating From the 2 Regimens

### Pregnancy Outcomes in High LH Level or High Testerone Level Patients

There were 5 cases presenting baseline LH levels >10 IU/L (11.33, 11.98, 21.78, 13.2, and 13.71 IU/L) in the study group. These patients had high proportions of metaphase II (MII) oocytes (22/28, 26/26, 8/17, 17/27, and 22/28, respectively) and 4 out of 5 patients achieved ongoing pregnancy. Fifteen patients presenting baseline testerone levels >0.57 ng/mL have similar mature oocyte retrived (15.2 ± 4.15) and ongoing pregnancy rates (12/15) when compared with normal T level patients in the study group.

Ten patients presenting high baseline LH levels and 16 patients presenting high baseline T levels in the control group, their mature oocyte retrived (17.9 ± 9.35, 16.57 ± 7.81) and ongoing pregnancy rates (8/10, 10/16) showed no difference when compared with those with normal levels.

### Logistic Regression of Pregnancy Outcome in Patients With PCOS

Logistic regression with the dependent variable ongoing pregnancy, and independent variables the baseline characteristics of patients recruited and type of stimulation protocol, revealed a significant negative effect of duration of infertility on the outcome (Table 4).

**TABLE 4:**
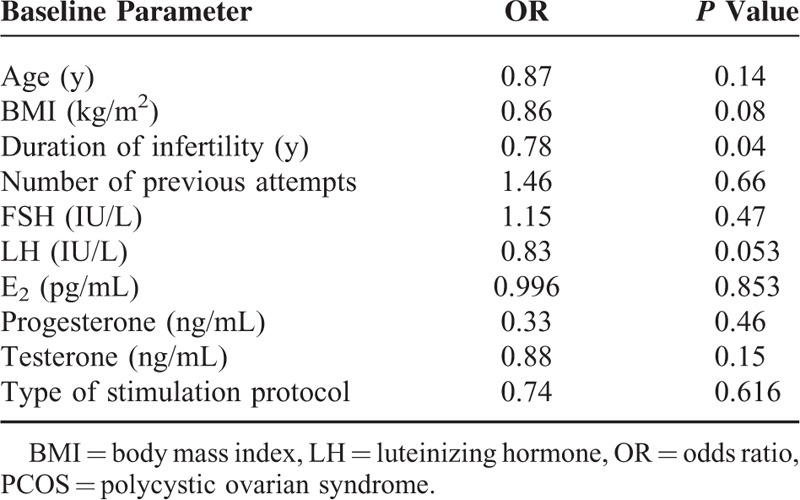
Logistic Regression of Pregnancy Outcome in Patients With PCOS

## DISCUSSION

Our results showed better IVF results in the MPA group. As most confounding variables were not significantly different between the 2 groups, and those that were significantly different had better values in the MPA group, we can conclude that the difference in outcomes was not due to confounding variables, but more likely due to the efficacy of the MPA cotreatment with hMG protocol in PCOS patients undergoing COH for IVF. To our knowledge, no studies have reported using combination of hMG and MPA as a controlled ovarian stimulation regimen in IVF cycles for women with PCOS.

The proportion of mature oocytes per retrieval, the clinical pregnancy rate per transfer, implantation rate, and cumulative pregnancy rate per patient in the study group are comparable to the short protocol. Moreover, the fertilization rate and the ongoing pregnant rate in the study group were higher than that in the short protocol. These results support that supplementation of MPA in COH is effective and feasible, without deteriorating the pregnancy outcomes. In addition, the total amount of gonadotrophin units required was significantly higher in the MPA group compared with the short protocol. The action of progestin inhibiting ovulation was widely used in research of oral contraception pills. Previous studies showed that the duration of gonadotropin administration and the hMG dose were higher in the usage of oral contraceptive pill (OCP) pretreatment in GnRH antagonist cycles.^17^ Follicle becomes less sensitive to gonadotropin stimulation in the high progesterone environment.^5^ One possible reason for the higher total amount of gonadotrophin units in the MPA group is associated with the pituitary suppression during the ovarian hyperstimulation.

Previous studies provide the foundation of the novel usage of MPA in PCOS patients. Progestin may have multiple roles in oocyte health and meiosis as well as cytoplasmic maturation and fertilization during the periovulatory interval in primates.^18^ Progestin replacement prevented oocyte degeneration and increased the percentage of mature, MI oocytes recovered. Replacement of the ovulatory bolus of hCG with progestin in vivo yielded a greater proportion of mature oocytes capable of fertilization and cleavage in vitro relative to vehicle.^18^ Resumption of meiosis in oocytes is triggered by steroid hormones, specifically progestin, in certain species.^19^ The biological action of progestin are mediated by genomic isoforms of progestin receptors (PR) and PR was identified in granulosa cells.^20^ In human and rhesus monkeys, high ratios of progestin to E_2_ in follicular fluid were associated with better embryo development,^21^ whereas the exact mechanisms of progestin that contributes to oocyte maturation and competence in human should be explored.

The effect of progestin administration on the hypothalamic-pituitary-ovarian (HPO) axis for premature LH surge suppression and premature ovulation remains to be explored. Progestin can block the estradiol-induced LH surge by preventing the “activation” of the GnRH surge induction system by estradiol.^22^ We chose 10 mg MPA because previous studies indicated that MPA 10 mg can be used to inhibit ovulation, whereas MPA 5 mg failed to inhibit ovulation.^23^ In this trial, MPA can block premature LH surge after a 5-day administration, which is agreed with our previous studies.^6^ Previous studies also demonstrated that progestin, administered during the normal follicular phase, slowed LH pulse frequency, augmented pulse amplitude, and reduced mean plasma LH levels compared to those in untreated women.^24^ The mechanisms of action of progestin on pituitary LH and FSH discharges are mediated by the interaction estradiol, progestin, and the GnRH neurosecretory system.^25,26^ There was a trend of progressive deeper serum LH suppression during the hMG and MPA protocol. It could contribute to the higher amount of hMG used, and both protocols used in this study worked well with regard to prevention of a premature LH surge. We did not use OCP pretreatment and found that high baseline LH and testerone levels did not appear to have adverse effect on the oocytes and embryos originated from the 2 groups.

Another highlight of this study is the low incidence of OHSS. Although avoidance of OHSS is paramount to patient safety, the ideal management of PCOS patients would use a treatment that minimizes the patient's risk while achieving optimal IVF cycle outcomes. In the present study, it was shown that the MPA protocol was associated with a low probability of moderate or severe OHSS. The MPA group included 41 women who had ≥20 follicles of ≥10 mm on the day of the triggering oocyte maturation with 0.1 mg Decapeptyl and 1000 IU hCG injection. The average number of oocytes collected was 15.28 ± 7.89, and there were no cases of early onset OHSS of any degree. The short group included 43 women who had ≥20 follicles of ≥10 mm on the day of the triggering oocyte maturation with 2000 IU hCG injection. The average number of oocytes collected was 15.8 ± 8.49. Despite the fact that the subjects in the short protocols are also high responders, only 2 cases were diagnosed as early-onset moderate OHSS, which may be associated with the lower trigger dose of 2000 IU hCG.

It is not, however, clear what is the basis of the low occurrence of OHSS observed in the MPA group. Even of the women who produced >20 oocytes (n = 41), no one complained of abdominal distension, nausea, or vomiting during ovarian stimulation. Progestin prevents incidence of OHSS in controlled ovarian stimulation at both follicular phase and luteal phase.^5,6^ Despite the lower dose of hCG triggering and freeze-all strategy, other factors related to the role of the high progestin milieu may also be associated with the low incidence of OHSS. One explanation is that exogenous progestin may exert an autoregulatory positive feedback action to enhance the production of endogenous progestin. It has been demonstrated in rat granulosa cells that progestins can stimulate progestin biosynthesis via enhancing the 3β-hydroxysteroid dehydrogenase (3β-HSD) enzyme.^27–29^ However, any benefit of this protocol in such a context needs to be further investigated.

Duration of infertility was shown to have a negative effect on ongoing pregnancy rates, as calculated by logistic regression. Possible explanations for the negative association of duration of infertility with pregnancy rates in our study are: those patients with long duration of infertility may have history of treatment failure. Previous studies reported that lower pregnancy rates were associated with high BMI in patients who had been treated with either agonist^30^ or antagonist protocols,^12,31^ but this was not in line with our study. Possible reason for this may be: compared with those women in American, PCOS patients in China have lower BMI. Despite this, however, neither high baseline LH nor T has any effect on the number of COCs retrieved and ongoing pregnancy rates, as shown in our study.

We used GnRHα and a low dose of hCG (1000 IU) to cotrigger in order to avoid a low response of the HPO axis. There were 6 cases that did not present a sufficient response (LH < 20 IU/L) after cotrigger by GnRHa and hCG. Their oocyte retrieval rates and proportion of MII oocytes were similar to the short protocol with sufficient response. So we deduce cotrigger by GnRHa and a low dose of hCG had a favorable effect on oocyte maturation in this protocol.

MPA shows no detrimental role on oocytes and embryos development potential in the normal ovulating women in our previous reports.^6^ We had followed up 587 live-born infants born from luteal-phase ovarian stimulation and found that high progestin status in COH did not increase the risk of live-birth defects compared with conventional ovarian stimulation.^32^ In this study, MPA cotreatment with hMG did not damage oocyte quality, but elevated the fertilization rate and development potential of embryos in PCOS patients.

FET has been widely used recently, with the safety of cryopreservation techniques being confirmed, as well as the advantages of the “freeze-all” strategy, which can increase cumulative pregnancy rates, decrease multiple pregnancy rates and ectopic pregnancy rates, and reduce the risk of OHSS.^9,32,33^ But whether the freeze all policy is the way to go in reproductive medicine is still for debate. FET singletons have a higher mean birthweight than singletons born after transfer of fresh embryos, and FET singletons are at an increased risk of being born large for gestational age (LGA).^35^ A risk increase of being born LGA in FET singletons remained even after adjusting for parity in the complete sibling cohort and after adjusting for birth order. This indicates that the freezing and thawing procedures may play an independent role for the growth potential of the fetus. This may be due to epigenetic alterations at the early embryonic stages during freezing/thawing.^35^ As LGA in human singletons from FET transfer is not related to an increased risk of malformations, there is no reason to assume that the higher risk of LGA in FET singletons mirrors the “large offspring syndrome” observed in animals.^35–38^

The trial limitation is not using random number table based on a computer-generated drawing of numbers. Using alternative numbering in allocation does not completely control the possible interferential factors between the 2 groups. Our trial strictly executed according to the requirement of good clinical practice (GCP) guidelines. The blinded RCT will be further planned to address the relevant issue in the near future.

In conclusion, the administration of MPA in COH decrease the incidence of premature LH surge without adversely affecting the pregnancy outcome for IVF/ICSI patients with PCOS, the new treatment provides a novel sight to overcome premature ovulation and decrease the incidence of OHSS for patients with PCOS. The role of MPA in COH appears to be promising although many questions about the extent of progestin priming and the possible influence on embryo development potential remain. Further study will be needed to extend sample size and follow up the long-term safety for children.
